# Germination-Induced Changes in the Nutritional, Bioactive, and Digestive Properties of Lima Bean (*Phaseolus lunatus* L.)

**DOI:** 10.3390/foods14122123

**Published:** 2025-06-17

**Authors:** Yingjinzhu Wu, Weon-Sun Shin

**Affiliations:** Department of Food and Nutrition, College of Human Ecology, Hanyang University, 222 Wangsimni-ro, Seoul 04763, Republic of Korea; wuyingjinzhu@gmail.com

**Keywords:** germination, lima bean, antinutrient, antioxidant activities, limited proteolysis profile

## Abstract

(1) Background: Lima beans (*Phaseolus lunatus* L.) are underutilized legumes rich in nutrients; however, they are limited by the presence of antinutritional content. In this study, we evaluated the effects of a low-cost germination treatment on the nutritional composition, antinutrient content, and digestibility of whole lima beans. (2) Methods: unlike previous studies focused on common legumes or isolated proteins, this work adopted a whole-seed approach and integrated multiple parameters to provide a comprehensive evaluation. (3) Results: The total polyphenol and flavonoid contents increased significantly, by 215.57 mg GAE/g and 71.84 mg RE/g, respectively, at 72 h of germination (*p* < 0.05). Antioxidant activity nearly doubled compared to raw beans, while the tannins and phytic acid content decreased significantly (*p* < 0.05). SDS-PAGE showed that germination enhanced digestibility by breaking down high-molecular-weight proteins into smaller fragments (15–30 kDa). Notably, samples germinated for 12–48 h showed higher digestibility after 2–3 h of limited proteolysis. (4) Conclusions: these findings indicate that germination effectively reduces antinutritional factors and improves digestibility, making processed lima beans a promising nutrient-dense ingredient for food formulations.

## 1. Introduction

Legumes are the second most important food source, after cereals [1]. They provide vital nutrients such as minerals, carbohydrates, vitamins, and dietary fiber and serve as an affordable and sustainable source of plant-based proteins compared to animal-derived products [2]. Legumes are broadly categorized into major and minor species [3]. Minor legumes, such as lima bean, winged bean, marama bean, and Bambara groundnut, are often considered neglected, underutilized, and underexploited. In contrast, major legumes, such as soybean, cowpea, and black soybean are widely cultivated and well-researched. Despite their limited use, minor legumes hold considerable potential to enhance livelihoods through the diversification of cropping systems, improved nutritional profiles, strengthened food security, expanded market opportunities, and greater ecosystem resilience [4]. Additionally, they offer promising alternatives for cultivation under challenging environmental conditions.

Lima beans (*Phaseolus lunatus* L.), native to Latin America and Central America, widely cultivated in both tropical and temperate regions, are an underutilized legume with significant agronomic and nutritional value [5]. Lima beans are rich in protein (8.61–26.02%), carbohydrates (50.44–77.39%), and dietary fiber while being low in fat (0.21–3.12%) [2]. They also provide essential vitamins, including thiamine, riboflavin, niacin, and vitamin B6, which function as coenzymes in macronutrient metabolism [6]. Moreover, studies have shown that lima beans possess gastroprotective, antimicrobial, antioxidant, antiviral, and antihypertensive properties [7]. However, their consumption remains limited due to the presence of naturally occurring antinutritional factors (ANFs) such as trypsin inhibitors, phytic acid, tannins, oxalates, and cyanogenic glycosides [8]. These compounds can reduce the mineral bioavailability, impair protein digestibility, and decrease the overall food quality.

Therefore, reducing or eliminating ANFs through processing methods is essential to enhance the nutritional quality and acceptability of lima beans [9]. Processing techniques such as fermentation, dehulling, cooking, soaking, and germination are widely employed to mitigate these factors [10,11,12]. Germination is a cost-effective and natural bioprocess that enhances nutrient bioavailability and reduces ANFs through enzymatic degradation, increased metabolic activity, and the synthesis of bioactive compounds [8]. Germination has been shown to reduce phytates and tannins, improve the content and digestibility of proteins, and enhance the antioxidant activity and mineral bioavailability in various legumes including mung beans, white kidney beans, and pigeon peas. Although research on germinated lima beans remains limited, several recent studies provide encouraging evidence. Adebayo and Okoli reported that 5 days of germination significantly increased the protein and mineral contents in lima beans [8]. Similarly, another study showed that 72 h of germination improved the in vitro protein digestibility by 14.8%, antioxidant activity by 33.5%, and reduced antinutritional factors such as phytic acid, tannins, and trypsin inhibitors [2]. These findings suggest that germination may indeed be a promising strategy to unlock the full nutritional potential of lima beans—supporting the rationale for the further investigation of this underutilized legume.

However, germination can sometimes have negative effects as well. Some reports have indicated that prolonged germination can result in the loss of heat-sensitive vitamins or phenolic compounds and may cause undesirable changes in taste, color, or texture [13]. Moreover, excessive germinating could impair functional properties such as solubility or emulsification due to the over-degradation of macromolecules [14]. These variations highlight the importance of optimizing the germination time to balance nutritional improvements with sensory and technological quality, ensuring the suitability of germinated legumes for food applications.

Compared to other legumes, the utilization of lima beans remains relatively limited, which may be attributed to a lack of awareness regarding their nutritional and health benefits, as well as optimal preparation methods. Due to their mild flavor, lima beans are incorporated into a variety of culinary applications [15]. Traditionally, lima beans are cooked prior to consumption and can be prepared in diverse forms, including fresh, sautéed, boiled, canned, frozen, baked, dried, or roasted [16]. In addition to their culinary versatility, lima beans are a potential source of native and oxidized starches, which may support the food industry in meeting increasing starch demands. Moreover, they possess reported medicinal properties [17]. Despite their potential, the hard-to-cook nature of lima bean seeds remains a barrier to broader utilization. However, appropriate processing methods can significantly improve their functional properties and consumer acceptance. The commercialization of underutilized crops like lima beans plays a crucial role in enhancing food security, improving the nutritional status, increasing the crop diversity and yield, and alleviating poverty, particularly in developing regions.

In this study, we aimed to comprehensively evaluate the effects of germination on the nutritional composition, antioxidant activities, and ANFs of lima beans and their bioactive potential. By analyzing these changes, we seek to provide a scientific foundation for enhancing the utilization of germinated lima beans in food applications and promoting the broader adoption of underutilized legumes in global diets.

## 2. Materials and Methods

### 2.1. Materials

The raw lima beans used in the experiment were sourced from Peru and procured from the Korean Naver website in June 2024. Chemicals and reagents, including Brilliant Blue G/R, Albumin, 2 N Folin–Ciocalteu, sodium carbonate (Na_2_CO_3_), Gallic acid, 2,2-diphenyl-1-picrylhydra-zyl (DPPH), 2,2′-Aziono-bis (3-ethylbenzthiazoline-6-sulfonic acid)(ABTS), trichloroacetic acid, pepsin from porcine gastric mucosa (powder, ≥400 units/mg protein), trypsin from bovine pancreas (powder, ≥6000 BAEE units/mg protein), tetramethylethylenediamine (TEMED), and tannic acid were purchased from Sigma Aldrich Co. (St. Louis, MO, USA). Phenol, sodium hydroxide (NaOH), acetic acid, ammonium persulfate, and ethanol were purchased from Samchun Chemical Co. (Seoul, Republic of Korea). Sulfuric acid, 90% diethylene glycol, potassium ferricyanide (K_3_Fe(CN)_6_), and ferric chloride were obtained from Daejung Chemical Co. (Siheung, Gyeonggi-do, Republic of Korea). Notably, 30% acrylamide solution and 4× reducing sample buffer were obtained from Gen DEPOT Co. (Barker, TX, USA). In addition, 1.5 M Tris-HCl (pH 8.8), 1.0 M Tris-HCl (pH 6.8), glycine, methanol, and phytic acid assay kits were purchased from BIosesang Co. (Yongin, Gyeonggi-do, Republic of Korea), Phygene Scientific Co. (Fuzhou, Fujian, China), JUNSEI Chemical Co. (Tokyo, Japan), Supelco Co. (Bellefonte, PA, USA), and Megazyme Co. (Bray, County Wicklow, Ireland). All chemical reagents were above the guaranteed grade.

### 2.2. Germination Process of Lima Bean

**Figure 1:**
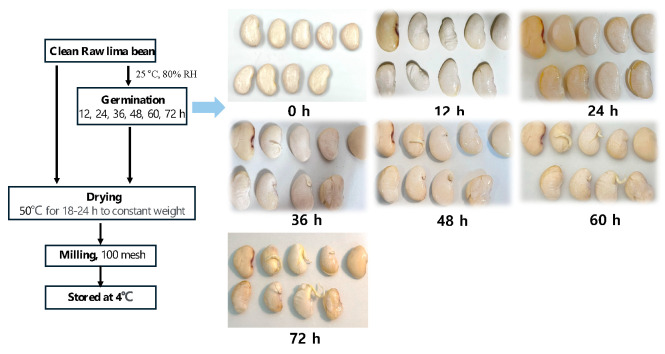
Preparation process and appearance changes of germinated lima beans—0–72 h: germinated lima beans during 0, 12, 24, 36, 48, 60, and 72 h.

Germination of lima bean seeds was performed as described previously, with slight modifications [18,19]. Clean beans were placed in a germination container to maintain a stable room temperature and humidity of 25 °C and 80%, respectively. Distilled water was added in a controlled manner not to cover the beans. The beans were then covered with damp cotton to maintain humidity and appropriate temperature while preventing direct sunlight exposure for 72 h. The seeds were sprayed with 50 mL of distilled water every 12 h to keep them moist and active and prevent mold growth (Figure 1). After germination, the seeds were dried in a hot air oven (ModelB35E, Apex Scientific Ltd., London, UK) at 50 °C until they reached a constant weight. The dried seeds were sorted by removing dirt and broken beans. Clean beans (100 g) were milled into powder, sieved through a 100-mesh sieve, packed in polythene bags, and stored in a refrigerator until use. The experimental groups were categorized based on germination duration: 12 (G12), 24 (G24), 36 (G36), 48 (G48), 60 (G60), and 72 h (G72). Raw lima beans (G0) were used as the control group.

### 2.3. Visual Characteristics of Germinated Lima Bean

The length of the sample was quantified using advanced research methods [20]. The lengths of 10 randomly selected lima bean seeds from each experimental group were measured with a digimatic caliper (Mitutoyo, Korea) with a precision of ±0.01 mm.

The germination rate of lima beans was tested using 100 seeds in three replicates (according to the method described in Section 2.2) [21]. The germination rate was calculated as the number of germinated seeds at each time interval (0–72 h, 12 h intervals) divided by the total number of seeds. The experimental design of the germination test was completely randomized and repeated three times.

### 2.4. Compositions of Germinated Lima Bean

#### 2.4.1. Protein Concentration (PC)

The protein concentration in the samples was quantified using the Bradford method [22]. Notably, 100 mg of the powder obtained in Section 2.2 was weighed and completely dissolved in 1 mL of distilled water. Thereafter, 0.1 mL of the sample solution was added to 5 mL of Bradford reagent. After allowing the reaction to stabilize for 5 min, absorbance was measured at 595 nm using a UV-visible spectrophotometer (Genesys 10S; Thermo Fisher, Waltham, MA, USA). A standard curve was prepared using bovine serum albumin as the standard protein to calculate the protein concentration of the samples.

#### 2.4.2. Total Carbohydrate Content (TCC)

Total carbohydrate content was determined using the phenol–sulfuric acid method [23,24]. The sample was diluted at a 1:2000 (*w*/*v*) ratio in distilled water and mixed with 5 mL of sulfuric acid. The mixture was then incubated at 90 °C for 15 min. Next, 1 mL of phenol was added and mixed thoroughly. Then, the absorbance was measured at 490 nm using a UV-visible spectrophotometer. Glucose was used as the standard.

#### 2.4.3. Total Polyphenol Content (TPC)

Total polyphenol content was determined using the Folin–Ciocalteu method [25]. To measure TPC, 150 μL of the sample solution, 2400 μL of distilled water, and 50 μL of 2 N Folin–Ciocalteu reagent were mixed and incubated for 3 min. After incubation, 300 μL of 5% Na_2_CO_3_ was mixed with the reaction mixture and incubated for 2 h in the dark. Absorbance was then measured at 725 nm using an ultraviolet UV-visible spectrophotometer. The TPC was calculated and expressed as milligrams of gallic acid equivalents per gram of dry weight (mg GAE/g dry weight), where GAE refers to the amount of gallic acid used as the standard compound to quantify total polyphenols.

#### 2.4.4. Total Flavonoid Content (TFC)

Total flavonoid content was determined using the Davis method [26]. To measure TFC, 100 μL of the sample solution, 1000 μL of 90% diethylene glycol, and 100 μL of 4% NaOH were mixed and then incubated for 1 h in a water bath at 37 °C. Absorbance was measured at 420 nm using a UV-visible spectrophotometer. The TFC was expressed as milligrams of rutin equivalents per gram of dry weight (mg RE/g dry weight), with RE indicating the amount of rutin equivalent to the measured flavonoid content in the sample.

### 2.5. Antinutrient Content

#### 2.5.1. Tannic Content (TC)

Tannic content was determined using the Folin–Ciocalteu method [27]. About 0.1 mL of the sample solution was added to a 10 mL volumetric flask containing 7.5 mL of distilled water, 0.5 mL of Folin–Ciocalteu phenol reagent, and 1 mL of 35% sodium carbonate solution. The volume was then adjusted to 10 mL with distilled water. The mixture was thoroughly shaken and maintained at room temperature for 30 min. A set of reference standard solutions of tannic acid (20, 40, 60, 80, and 100 µg/mL) was prepared in the same manner described earlier. Absorbances of the samples and standard solutions were measured against a blank sample at 700 nm using a UV-visible spectrophotometer. The TC was estimated in triplicates and expressed in milligrams of tannic acid equivalents per gram of dried sample.

#### 2.5.2. Phytic Acid Content (PAC)

Phytic acid content in the samples was measured using an enzymatic method with a phytic acid assay kit (K-PHYT) from Megazyme [28]. A calibration curve was constructed using standard phosphorus to quantify PAC according to the instructions in the assay kit.

### 2.6. Antioxidant Activities

#### 2.6.1. DPPH Radical Scavenging Activity

The radical scavenging activity of DPPH in the sample solution was determined following the Shafay method [29]. The sample solution and DPPH solution were stirred at a ratio of 3:1 and incubated in the dark for 30 min. Absorbance was measured at 517 nm using a UV-visible spectrophotometer. The DPPH free radical scavenging activity was calculated using Equation (1).

(1) $$DPPH\;free\;radical\;scavenging\;activity\;\left(\%\right)=[1-\frac{A_{sample}-A_{sample\;blank}}{A_{Control}}]\times 100$$

#### 2.6.2. ABTS Radical Scavenging Activity

The ABTS radical scavenging activity was determined using a previously proposed method [30]. Prior to the assay, 900 µL of ABTS^+^ solution was mixed with 100 µL of the sample solution. Absorbance was measured at 734 nm using a UV-visible spectrophotometer. The ABTS free radical scavenging activity was calculated using Equation (2).

(2) $$ABTS\;\;free\;radical\;scavenging\;activity\;\left(\%\right)=[1-\frac{A_{sample}-A_{sample\;blank}}{A_{Control}}]\times 100$$

#### 2.6.3. Reducing Power (RP)

The reducing power was measured according to Silva’s method [31]. A total of 1 mL of the sample solution was mixed with 1 mL of 0.2 M sodium phosphate buffer (pH 6.6) and 1 mL of 1% K_3_Fe(CN)_6_. The mixture was incubated in a water bath for 20 min. Then, 1 mL of 10% trichloroacetic acid (TCA: CCl3COOH, *w*/*v*) was added and the sample was centrifuged at 3000× *g* for 10 min (Model Combi 514R, Hanil Scientific Industrial Co., Ltd., Gimpo, Republic of Korea). The supernatant was collected (1 mL). After mixing with 5 mL of distilled water, 0.2 mL of 0.1% ferric chloride was added, and absorbance was measured at 700 nm.

### 2.7. In Vitro Limited Proteolysis Profile

In vitro limited proteolysis of proteins was determined using successive pepsin–trypsin enzyme systems, according to a previously described method of advanced research with minor modifications [32,33]. For pepsin digestion, in a 50 mL centrifuge tube, 0.5 g of protein sample was suspended in 9.5 mL of 0.1 M HCl and mixed with 5 mg pepsin in 0.5 mL of 0.1 M HCl. The mixture was gently shaken at 37 °C for 120 min. Then, the solution was neutralized with 1.0 M phosphate buffer (pH 8.0), followed by adding appropriate trypsin (100:1 ratio of substrate/enzyme ratio, *w*/*w*). The tubes were covered and incubated again at 37 °C for 120 min. The digestion fluid was collected hourly and heated at 95 °C to inactivate the enzymes. After centrifugation at 4000× *g*, the supernatant was collected for subsequent experiments. Digestibility was calculated using Equation (3).

(3) $$Digestibility(\%)=(1-\frac{P_{h}-P_{b}}{P_{s}})\times 100$$

The P_h_ and P_b_ denote the protein content in the digestion fluid and blank, respectively. P_s_ represents the protein content in the aliquot collected at time 0 when the protein concentrate was redissolved in the solution prior to the digestion process.

#### Sodium Dodecyl Sulfate Polyacrylamide Gel Electrophoresis (SDS-PAGE)

The SDS-PAGE was performed using a separation gel concentration of 12.5% and a stacking gel concentration of 4.5% [34]. The protein concentrations of all the samples were adjusted to 0.2 mg/mL. A volume of 30 μL of the sample was mixed with 10 μL of 4× reducing sample buffer (Tris-HCl 250 mM pH 6.6, SDS 8%, Glycerol 40%, Beta-mercaptoethanol 8%, Bromophenol blue 0.02%), heated in a boiling water bath for 5 min, and then cooled down before loading. The sample and marker loading volume were 10 μL and 5 μL, respectively. The gel was operated at a constant current of 40 mA for 2 h. After completion, the gel was stained with Coomassie brilliant blue solution (Coomassie brilliant blue 1% *w*/*v*, methanol 30% *v*/*v*, acetic acid 10% *v*/*v*, and distilled water 60% *v*/*v*) for 1 h and then destained overnight.

Densitometric quantification of SDS-PAGE bands was completed using ImageJ software (Version 1.53t, National Institutes of Health, Bethesda, MD, USA; https://imagej.net/ij/). Integrated density (IntDen) values were calculated based on fixed rectangular areas for three molecular weight ranges (75 kDa, 15–30 kDa, and <15 kDa) across different germination time points. Data are expressed in arbitrary units.

### 2.8. Statistical Analyses

All experiments in this study were performed in triplicate replicates and results were presented as the mean ± standard deviation. Data analysis was carried out using SPSS 26 (SPSS Inc., Chicago, IL, USA). Analysis of variance was performed, followed by Duncan’s post-test to assess significance with a 95% confidence interval. A heat map was used to show the significance of Pearson’s correlation with the experimental results. The heat map and graph visualization were performed using the Origin program 2021 (OriginLab Co. Northampton, MA, USA).

## 3. Results and Discussion

### 3.1. Visual Characteristics of Germinated Lima Bean

Table 1 presents the changes in the length and germination rate of lima beans during the 0–72 h germination period. The initial average length of the raw lima beans (G0) was 2.44 cm. As germination progressed, the seed length increased significantly (*p* < 0.05); however, growth slowed after 48 h and minimal changes were observed between 48 and 72 h. During the early and middle stages of germination (G0–24), the seeds primarily absorb water and enter the preparatory phase, resulting in a relatively low germination rate. A rapid increase in germination was observed between 36 and 48 h (33.5–51.2%), stabilizing around 60–72 h (52.12–52.15%). These findings suggested that the water uptake during the initial phase promotes seed expansion. However, excessive or insufficient water may negatively affect the germination rate and seed length growth [19].

During germination, legume seeds undergo a complex series of physiological and structural changes that not only promote the water uptake, but also significantly increase tissue expansion. The main mechanism of this expansion is the enzymatic degradation of cell wall polysaccharides (e.g., pectin, cellulose, and hemicellulose) by endogenous enzymes, resulting in cell wall relaxation and increased cell extensibility [35]. At the same time, germination activates hydrolase systems that accelerate the breakdown of storage macromolecules including starch and protein. In addition, partial proteolysis during germination exposes hydrophilic residues, increases protein solubility, and promotes colloidal expansion [36]. In summary, these biochemical and structural changes are key mechanisms driving seed expansion, beyond simple hydration, and lay a key foundation for improving the nutritional function of germinating legumes. These changes have also been reported in lima beans specifically, where sprouting was shown to enhance the proteolytic activity and protein content [9], supporting the role of endogenous enzyme systems in driving biochemical transformation. Similarly, Shimelis and Rakshit demonstrated that the germination of legumes, including lima beans, enhances the enzymatic degradation of macromolecules and promotes nutritional improvement [37]. In summary, these biochemical and structural changes are key mechanisms driving seed expansion, beyond simple hydration, and lay a key foundation for improving the nutritional function of germinating legumes.

### 3.2. Compositions of Germinated Lima Bean

#### 3.2.1. Protein Concentration (PC)

Table 2 shows the changes in the composition of lima beans during the 0–72 h germination period. The PC of G0 was 10.08 µg/mL, which decreased to 8.38 µg/mL after 72 h of germination. The protein content decreased significantly as the germination time increased (*p* < 0.05). Similarly, Martin Rehman’s studies observed a decrease in PC and an increase in protein digestibility in chickpeas and lentils after soaking [38,39]. The reduction in PC during germination is likely due to the activation of proteases, which leads to the gradual breakdown of storage proteins into smaller peptides and free amino acids, providing essential nutrients for germ growth. As germination progresses, nitrogenous compounds are mobilized and redirected toward the synthesis of essential biomolecules, including metabolic enzymes, transport proteins, and nucleic acids, potentially leading to a decrease in the measurable protein content. Furthermore, the degradation of compact storage protein structures may induce changes in the solubility and molecular conformation, further contributing to the observed reduction in the protein concentration [40].

Although direct free amino acids (FAAs) measurements were not performed in this study, previous research on lentils and other legumes demonstrates significant increases in free amino acid levels during germination. Kuo et al. reported that lentil and pea seeds germinated for 2–6 days and observed a substantial accumulation of both free amino acids, mainly resulting from the proteolytic hydrolysis of storage proteins, and free non-protein amino acids, which are metabolites involved in various physiological processes, under light conditions using HPLC analysis [41]. Similarly, studies on Canadian lentil varieties reported 4- to 6-fold increases in free amino acid and peptide fragments after 4–6 days of germination, alongside the degradation of storage globulins [42]. Based on these findings, we speculate that the reduction in the protein concentration observed during lima bean germination may be caused by the proteolytic hydrolysis of storage proteins into smaller peptides and FAAs, which could serve as essential nutrients for seedling growth.

#### 3.2.2. Total Carbohydrate Content (TCC)

In this study, germination did not significantly affect TCC (Table 2). During germination, stored polysaccharides are likely mobilized to support metabolic activities; however, it is possible that concurrent biosynthetic pathways help to maintain overall carbohydrate levels through sugar interconversion and redistribution. Another possibility is that the relatively short germination time or specific environmental conditions (e.g., temperature or humidity) used in this study were not sufficient to trigger substantial carbohydrate catabolism.

However, different results were observed in other studies, with lentil, soybean, and lupine showing a gradual decrease in their carbohydrate content with an increasing germination time [43]. Conversely, Farinde et al. (2018) found that there was an increase in the TCC across all processing treatments of lima beans, including germination [19]. Meanwhile, germinated lima beans exhibited the lowest TCC (59.23%) among all treated samples, which could be attributed to the breakdown of seed carbohydrates into simple sugars, which are the primary energy sources for embryo growth [44]. These discrepancies could arise from differences in germination protocols, seed varieties, or analytical methods used to quantify carbohydrates. Therefore, further research is needed to elucidate the specific metabolic responses of lima beans to germination under various conditions

#### 3.2.3. Total Polyphenol Content and Total Flavonoid Content (TPC and TFC)

The TPC and TFC of germinated cereals have been extensively studied to evaluate the antioxidant activity [45]. Table 2 shows the TPC (124.74–215.57 mg GAE/g) and TFC (58.16–71.84 mg RE/g) of germinated lima beans, both of which increased significantly with an extended germination time (*p* < 0.05). Germination significantly increases the phenolic content, thereby enhancing the antioxidant activity [45]. Similarly, in other studies on germinated lima beans, the highest TPC (139.74 mg GAE/100 g) and TFC (33.63 mg QE/100 g) were also observed at 72 h of germination [18]. Furthermore, a study on six legume species reported that after 5 d of germination, the phenolic content of mung beans, white cowpeas, soybeans, and peanuts increased by two-fold, whereas the TPC of black beans and adzuki beans increased by approximately 50% and 25%, respectively [46]. The increase in the TPC observed during germination may be explained by both the enhanced release of bound phenolic compounds over time and the de novo synthesis of polyphenols via metabolic pathways such as the shikimate, pentose phosphate, and phenylpropanoid pathways [47].

Furthermore, although the TFC in G72 was approximately 1.2 times higher than that in G0, the overall changes in flavonoid levels throughout the 0–72 h germination period were relatively limited. Similar increases in flavonoid accumulation during germination have also been reported in the studies by Wu et al. and Popoola, who observed an elevated TFC in germinated seeds of rice, *Phaseolus vulgaris, Pisum sativum*, and *Amaranthus viridis* [48,49]. This increase is likely related to the activation of key biosynthetic enzymes, such as chalcone isomerase and phenylalanine ammonia-lyase, which are known to play crucial roles in the flavonoid biosynthetic pathway [50].

Plants naturally produce phenolic compounds during growth and development as a defense mechanism against biotic stresses such as diseases, insects, and environmental stressors [51,52]. Substantial changes in the phytochemical composition during germination are considered a natural response of plants to environmental conditions.

### 3.3. Antioxidant Activities

Dietary antioxidants help combat reactive oxygen species and free radicals generated during the cellular metabolism or peroxidation of lipids and other biological molecules, resulting in reducing the risk of chronic diseases [53].

In this study, the antioxidant activity of germinated lima beans was assessed by evaluating their free radical scavenging activity using ABTS and DPPH assays, as well as their reducing power. As shown in Table 3, DPPH radical scavenging activity increased from 47.47% to 65.77% in G0 and G72, respectively, and the ABTS radical scavenging activity increased from 10.50% (G0) to 31.75% (G72). The reducing power increased from 0.50 (G0) to 0.70 (G72).

In a study on lima beans and adzuki beans, the antioxidant activity increased from 30.55% (RG) to 45.68% (G72) and from 28.77 (RG) to 39.25% (G72), respectively. Within 72 h of germination, the antioxidant activities of lima beans and adzuki beans increased by 49.52% and 36.42%, respectively [18]. The antioxidant compounds increased due to the amount of the phenolic content that increased in germinated legumes as a result of the presence of various hydroxyl groups that behaved like free radical scavengers and resulted in an increase in the antioxidant activity of germinated grains. In addition to phenolics, various bioactive compounds may also be synthesized or activated during germination, further contributing to the antioxidant potential [54]. Several studies have also reported that the accumulation of phenolic compounds during germination is attributed to the activation of hydrolytic enzymes, which degrade cell wall structures and promote the release of bound phenolics [55]. These secondary metabolites are produced as part of the plant’s adaptive response mechanisms to environmental stress factors encountered during germination [47]. In addition to enhancing antioxidant compounds, germination can also reduce potentially toxic or pro-oxidant components, such as phytic acid, tannins, and trypsin inhibitors [19]. These compounds, common in raw legumes, may impair nutrient absorption and contribute to oxidative stress. During germination, enzymes like phytase and polyphenol oxidase are activated, promoting the breakdown of these anti-nutritional factors into less harmful forms [56]. Combined with the increased synthesis of antioxidant phytochemicals, this contributes to the overall improvement in the nutritional and functional quality of germinated lima beans.

### 3.4. Anti-Nutritional Components (ANFs)

ANFs are naturally occurring compounds that interfere with the digestion and absorption of essential nutrients such as proteins and vitamins. In some cases, these compounds exert toxic effects or induce undesirable physiological responses including gastrointestinal discomfort and flatulence [57]. As a result, the bioavailability of minerals in cereal-based diets is often limited to just 5–15%, posing a significant nutritional challenge, particularly in developing countries [58]. Various techniques have been used in plant breeding to reduce the ANF effects, such as germination, which is a cost-effective method for enhancing the nutritional qualities and functional characteristics of grains [59].

In this study, germination significantly reduced ANF levels in lima beans (*p* < 0.05). The TC decreased from 1.58 mg/g (G0) to 0.26 mg/g (G72), while the PAC declined from 34.1 mg/g (G0) to 18.9 mg/g (G72) (*p* < 0.05) (Table 4). These results align with previous findings, which observed the decline trend of 42.62% and 68.85% in the PAC and TC in germinated kidney beans [60], respectively, and a 43% decline in the TC in chickpeas after 24 h of germination [61]. Similarly, the PAC of germinated chickpeas decreased by 57.35% [62].

The reduction in the PAC can be largely attributed to the activation of endogenous phytase enzymes during germination. Dong and Saneoka reported that phytase activity in germinating soybeans increased significantly from day 1 to day 4, coinciding with a marked decline in the phytic acid content and enhanced mineral bioavailability [63]. A similar enzymatic mechanism is likely at work in lima beans, where phytase hydrolyzes phytic acid into lower inositol phosphates with a weaker mineral chelation capacity and lower pro-oxidant potential [19]. In addition, the efficiency of phytic acid degradation is also influenced by the germination medium; greater phytate loss has been observed in distilled water compared to tap water, likely due to the absence of metal–phytate complexes that otherwise inhibit phytic acid diffusion [58].

Tannins, another group of polyphenolic ANFs, are high-molecular-weight compounds (≥500 Da) capable of precipitating proteins, thereby reducing protein bioavailability [64]. Tannin reduction during germination is also driven by multiple mechanisms, including enzymatic oxidation by polyphenol oxidase, hydrophobic interactions with proteins, and leaching into the soaking medium [65]. Although phytase and polyphenol oxidase activities were not directly measured in this study, the significant decreases in the PAC and TC strongly suggest active enzymatic degradation.

Other common antinutritional factors in legumes, such as trypsin inhibitors and lectins, were not included in this study. However, previous research has shown that these proteins are typically reduced during germination due to protease activity and thermal instability [65]. Future studies should include a broader range of ANFs and associated enzyme activity measurements to provide a more comprehensive evaluation of germination-induced nutritional improvements in lima beans.

### 3.5. Non-Reducing SDS-PAGE

**Figure 2:**
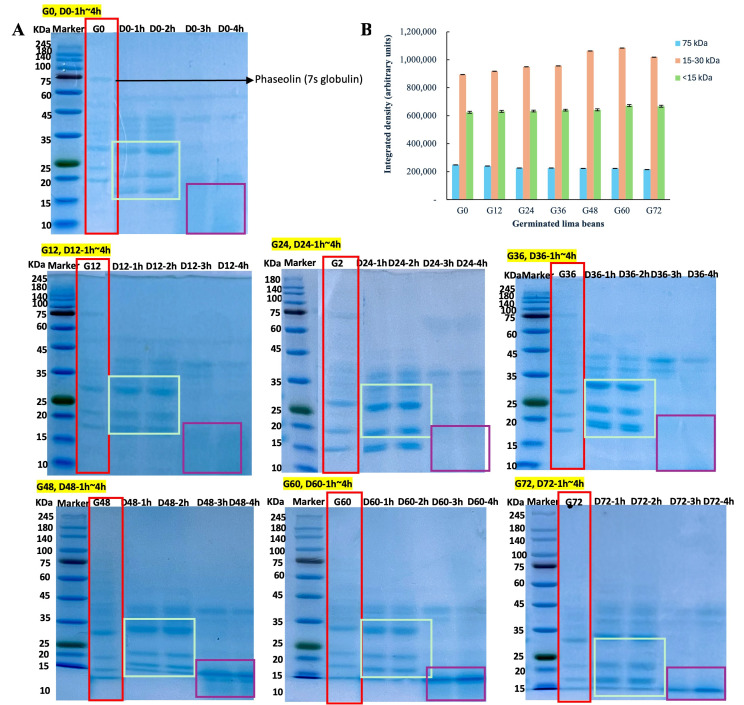
Non-reducing SDS-PAGE profiles and densitometric analysis of lima bean proteins during germination (0–72 h) under limited in vitro proteolysis. (**A**): Representative non-reducing SDS-PAGE gel images of lima bean proteins subjected to germination (0–72 h) and limited in vitro proteolysis (1–4 h). (**B**): Densitometric quantification of SDS-PAGE bands using ImageJ software. Integrated density (IntDen) values were calculated based on fixed rectangular areas for three molecular weight ranges (75 kDa, 15–30 kDa, and <15 kDa) across different germination time points. Data are expressed in arbitrary units.

The effects of germination (0–72 h) and limited in vitro proteolysis (1–4 h) on protein band patterns are presented in Figure 2A. Phaseolin, a major globulin storage protein, was primarily present at 75 kDa in the G0. The densitometric quantification of SDS-PAGE bands using ImageJ software IntDen values is shown in Figure 2B, where the 75 kDa band decreased from 249,683 at G0 to 223,633 at G48 and further to 215,753 at G72, indicating the progressive degradation of phaseolin with germination. However, with an increasing germination time, phaseolin progressively degrades into lower-molecular-weight proteins, particularly phaseolin subunit 3 (15–30 kDa). After germination (48–72 h), the 75 kDa band was nearly degraded, with more pronounced bands in the 15–30 kDa range, indicating further hydrolysis into smaller peptides.

As germination continued, some peptides were further degraded into fragments smaller than 15 kDa. These bands likely correspond to shorter polypeptides derived from the hydrolysis of phaseolin subunits. In support of this, the IntDen for the <15 kDa region showed a gradual increase from 624,089 at G0 to 672,074 at G60, remaining relatively high at 668,128 at G72. Specifically, they may include α-chain and β-chain fragments of phaseolin or residual peptides from vicilin-like proteins, as observed in other legume species undergoing similar proteolytic processes [66]. In addition, protease-resistant peptides originating from conserved regions of globulin proteins may also contribute to these low-molecular-weight bands. These low-molecular-weight peptides may include bioactive peptides with antioxidant or antihypertensive potential, which have been identified in other legume-derived hydrolysates [67]. However, partial digestion may expose allergenic peptides, particularly from storage proteins like phaseolin, a major IgE-binding allergen in the Phaseolus species, and vicilin or convicilin, whose hydrolysis fragments still retain an IgE-binding capacity [68,69].

The composition and proteolytic susceptibility of phaseolin vary across *Phaseolus* species. In *Phaseolus vulgaris* (common bean), phaseolin accounts for over 50% of the total seed protein [66]. In contrast, other reports showed that *Phaseolus lunatus* L. (lima bean) exhibits weaker phaseolin bands and greater resistance to enzymatic proteolysis [70], with protein bands predominantly appearing in the 20–25 kDa range during digestion [71].

Germination enhances the proteolysis of phaseolin, leading to its rapid degradation within 12–24 h of sprouting, which may be linked to a reduction in ANFs and improved protein digestibility [72]. During the 48–72 h germination period, the activation of endogenous proteases alters the protein structure, exposing previously hidden cleavage sites and making phaseolin more susceptible to pepsin and trypsin digestion [71].

These structural changes resulted in distinct low-molecular-weight protein bands following in vitro digestion, reflecting the production of peptides that were more accessible to the digestive enzymes. Consequently, germination for 48–72 h appears to be critical for enhancing the digestibility and bioavailability of phaseolin.

### 3.6. Digestibility of Germinated Lima Bean

**Figure 3:**
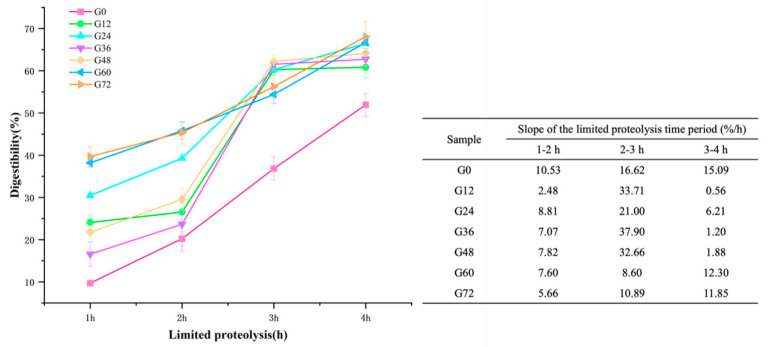
Digestibility of lima beans under different germination times with limited proteolysis (1–4 h).

Figure 3 shows the digestibility changes of germinated lima beans under limited proteolysis for 1–4 h. Notably, among all samples, G0 showed the lowest digestibility (9.70–51.94%) after 4 h of proteolysis, likely due to the presence of anti-nutritional factors that hinder protein digestion. During the first 1–2 h of proteolysis, the digestibility of G12–72 increased significantly (*p* < 0.05). However, between 2–3 h, the digestibility of G12 (26.56–60.27%), G24 (39.29–60.28%), G36 (23.63–61.43%), and G48 (29.57–62.23%) showed a nearly tripled increase in digestibility (*p* < 0.05). Notably, the hydrolysis of G12, G36, and G48 accelerated markedly during this period, with growth slopes of 33.71%/h, 37.90%/h, and 32.66%/h, respectively. This rapid increase can be attributed to enhanced trypsin activity, which effectively cleaves peptide bonds following the initial breakdown of protein structures by pepsin [73]. The sharp slopes observed at G12, G36, and G48 suggest that these germination stages created more accessible cleavage sites for trypsin, likely due to partial protein unfolding or a reduction in anti-nutritional factors. Pepsin and trypsin are the primary enzymes responsible for protein digestion and each targets specific amino acids [32]. Pepsin, active in acidic conditions, preferentially cleaves peptide bonds around aromatic amino acids such as phenylalanine, tyrosine, and tryptophan, which are present in lima bean storage proteins [74].

The significant increase in digestibility between 2–3 h of hydrolysis correlated with the breakdown of intermediate-molecular-weight peptides (30–50 kDa), which formed during the earlier stages of germination (12–36 h). The emergence of low-molecular-weight peptides (15–30 kDa) after G48–72 was consistent with the enhanced digestibility observed during the 3–4 h hydrolysis period. These peptides likely include hydrophilic oligopeptides and bioactive peptides that are readily absorbed by the gastrointestinal tract [75]. Such proteolysis not only facilitates peptide absorption, but also enhances the release and bioavailability of essential amino acids like lysine, leucine, and phenylalanine [18]. However, partial digestion may also transiently expose linear peptide sequences with allergenic potential, particularly from vicilin- or legumin-like proteins, as noted in the Phaseolus species [68,69]. These findings were supported by the SDS-PAGE results.

In the final hour of proteolysis, the increase in digestibility slowed, as indicated by the gentle slopes of G12 (0.56%), G24 (6.21%), G36 (1.20%), G48 (1.88%), G60 (12.30%), and G72 (11.85%). As hydrolysis progressed, the available peptide bonds that could be cleaved by the enzymes decreased, leaving behind smaller peptides that were more resistant to further enzymatic cleavage. This led to a slower rate of hydrolysis.

These findings align with previous studies showing significant increases in the in vitro protein digestibility of germinated legumes. Digestibility increased by 14.75% and 10.98% in germinated lima beans and adzuki beans, respectively [18]. Another study reported a 14.53% increase in the digestibility of *Phaseolus vulgaris*, whereas Sharma’s study observed an 11.62% increase in pigeon peas [76]. The enhancement in digestibility across various legumes is attributed to the reduction in anti-nutritional factors such as phytic acid and oligosaccharides. Furthermore, the breakdown of storage proteins into smaller peptides and amino acids enhances their digestibility and bioavailability [77].

### 3.7. Heat Map Representing the Pearson Correlation Between All Variables

**Figure 4:**
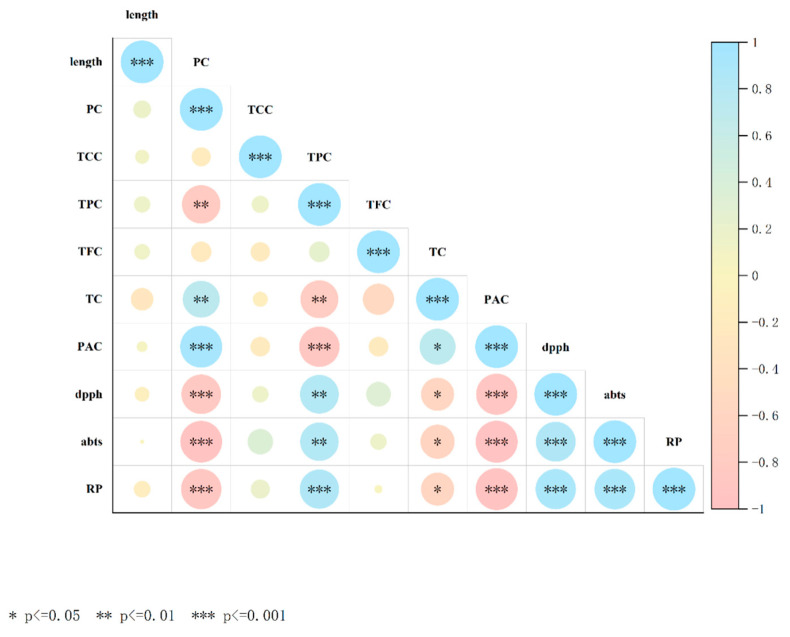
Heat map representing the Pearson correlation between all variables.

Pearson’s correlation analysis was performed to evaluate the inter-relationships between the variables in germinated lima bean seeds (Figure 4). The results showed a strong negative correlation between the PC and antioxidant activity (DPPH, ABST, and RP) (r between −0.80 and −0.94). In addition, the PC exhibited a significant negative correlation between the PAC and TPC and antioxidant activity (r between −0.88 and −0.96), indicating that as the germination time progressed, the PAC levels decreased while phenolic substances increased, resulting in an increased antioxidant capacity. The TPC and TFC showed a significant positive correlation with the antioxidant activity (r between 0.81 and 0.84), indicating that the phenolic content is an important contributor to the antioxidant capacity. Therefore, protein and active compound contents are directly related to the germination period.

## 4. Conclusions

This study demonstrated that germination significantly enhances the nutritional and functional properties of lima beans by inducing biochemical changes that improve digestibility, increase the TPC and TFC levels, and boost antioxidant activity, while effectively reducing ANFs such as phytic acid and tannins. The increases in the TPC and TFC can be attributed to the activation of metabolic pathways during germination, which stimulate the biosynthesis and release of these bioactive compounds. The antioxidant potential of the lima beans improved significantly, as reflected by the enhanced DPPH and ABTS radical scavenging activities.

Germination-induced enzymatic activity contributes to the degradation of complex macromolecules and facilitates the breakdown of protein and anti-nutritional compounds. The reduction in the phytic acid content may be linked to increased phytase activity, which hydrolyzes phytates and improves their mineral bioavailability. Regarding digestibility, limited proteolytic assays revealed that germination, particularly within the optimal period of 12–48 h, leads to significant improvements. The breakdown of high-molecular-weight proteins into smaller peptides enhances their accessibility to digestive enzymes, thereby increasing protein bioavailability.

These findings underscore the role of germination as a cost-effective and sustainable processing method to improve the nutritional quality of underutilized lima beans. Furthermore, germinated lima bean flour—due to its improved nutritional composition, enhanced protein digestibility, and reduced antinutritional factors—shows promise for incorporation into health-oriented food products such as high-protein snacks, composite flours for baking, and plant-based beverages. These applications could broaden the utilization of lima beans in functional and sustainable food systems, especially in plant-based and allergen-conscious formulations.

However, in this study, we focused on assessing the overall effects of germination on compositional, bioactive, and digestibility-related parameters from a whole-bean perspective. Instead, the processed samples were not evaluated in actual food systems, and assessments of their sensory attributes and processing performance are lacking. Therefore, further studies are warranted to explore the application potential of sprouted lima beans in the development of functional food products.

## Figures and Tables

**Table 1:** Visual characteristics of germinated lima bean.

	G0	G12	G24	G36	G48	G60	G72
**Length (cm)**	2.44 ± 0.05 ^d^	3.06 ± 0.19 ^c^	3.34 ± 0.154 ^b^	3.53 ± 0.15 ^a^	3.55 ± 0.16 ^a^	3.56 ± 0.16 ^a^	3.57 ± 0.16 ^a^
**Germination rate (%)**	0.00 ± 0.00 ^c^	0.00 ± 0.00 ^c^	0.00 ± 0.00 ^c^	33.05 ± 0.02 ^b^	51.20 ± 0.01 ^a^	52.12 ± 0.01 ^a^	52.15 ± 0.02 ^a^

G0–72: germinated lima beans during 0, 12, 24, 36, 48, 60, and 72 h; different letters (a–d) within the same column are showing significantly (*p* < 0.05).

**Table 2:** Compositions of germinated lima beans.

	G0	G12	G24	G36	G48	G60	G72
**PC** **(μg/mL)**	10.08 ± 0.08 ^a^	9.67 ± 0.16 ^b^	9.37 ± 0.08 ^bc^	9.20 ± 0.01 ^c^	9.08 ± 0.02 ^c^	8.55 ± 0.08 ^d^	8.38 ± 0.33 ^d^
**TCC** **(mg/g)**	88.4 ± 0.02 ^a^	88.7 ± 0.01 ^a^	88.3 ± 0.08 ^a^	88.6 ± 0.05 ^a^	88.8 ± 0.12 ^a^	88.4 ± 0.11 ^a^	88.8 ± 0.03 ^a^
**TPC** **(mg GAE/g)**	124.74 ± 0.17 ^c^	152.00 ± 24.13 ^bc^	169.14 ± 5.36 ^b^	171.52 ± 0.67 ^ab^	213.67 ± 37.12 ^a^	194.50 ± 3.87 ^ab^	215.57 ± 1.34 ^a^
**TFC** **(mg RE/g)**	58.16 ± 0.00 ^c^	58.42 ± 0.37 ^c^	62.19 ± 1.52 ^b^	62.50 ± 0.56 ^b^	62.90 ± 0.00 ^b^	63.95 ± 0.00 ^b^	71.84 ± 0.00 ^a^

G0–72: germinated lima beans during 0, 12, 24, 36, 48, 60, and 72 h; different letters (a–d) within the same column are showing significantly (*p* < 0.05).

**Table 3:** Antioxidant activities of germinated lima bean.

	G0	G12	G24	G36	G48	G60	G72
**DPPH radical** **scavenging** **activity** **(%)**	47.47 ± 0.29 ^e^	48.23 ± 0.61 ^e^	51.65 ± 2.75 ^d^	55.58 ± 1.73 ^c^	59.13 ± 1.98 ^b^	56.46 ± 2.96 ^bc^	65.77 ± 0.57 ^a^
**ABTS radical** **scavenging** **activity** **(%)**	10.50 ± 0.86 ^d^	12.34 ± 0.50 ^d^	16.67 ± 4.12 ^c^	19.92 ± 1.79 ^c^	24.21 ± 1.28 ^b^	31.04 ± 0.98 ^a^	31.75 ± 0.71 ^a^
**RP** **(O.D.)**	0.50 ± 0.05 ^c^	0.53 ± 0.01 ^c^	0.54 ± 0.00 ^c^	0.54 ± 0.00 ^c^	0.63 ± 0.02 ^b^	0.64 ± 0.02 ^b^	0.70 ± 0.01 ^a^

G0–72: germinated lima beans during 0, 12, 24, 36, 48, 60, and 72 h; different letters (a–e) within the same column are showing significantly (*p* < 0.05).

**Table 4:** Antinutrient content of lima bean at different germination times.

	G0	G12	G24	G36	G48	G60	G72
**TC** **(mg/g)**	1.58 ± 0.01 ^a^	0.46 ± 0.01 ^b^	0.34 ± 0.00 ^c^	0.32 ± 0.01 ^c^	0.30 ± 0.02 ^c^	0.28 ± 0.00 ^c^	0.26 ± 0.00 ^c^
**PAC** **(mg/g)**	34.1 ± 0.07 ^a^	31.6 ± 0.03 ^b^	29.2 ± 0.10 ^c^	26.1 ± 0.03 ^d^	23.4 ± 0.01 ^d^	22.5 ± 0.02 ^e^	18.9 ± 0.05 ^f^

G0–72: germinated lima beans during 0, 12, 24, 36, 48, 60, and 72 h; different letters (a–f) within the same column are showing significantly (*p* < 0.05).

## Data Availability

The original contributions presented in the study are included in the article, further inquiries can be directed to the corresponding author.

## Abbreviations

The following abbreviations are used in this manuscript:

G0-G72	Germinated lima beans during 0, 12, 24, 36, 48, 60, and 72 h
D0-D72 (1h–4h)	In vitro limited proteolysis with 1–4 h
PC	Protein concentration
TCC	Total carbohydrate content
TPC	Total polyphenol content
TFC	Total flavonoid content
TC	Tannin content
PAC	Phytic acid content
RP	Reducing power
SDS-PAGE	Sodium dodecyl sulfate–polyacrylamide gel electrophoresis
ANFs	Antinutritional factors
